# Targeting programmed cell death pathways: emerging therapeutic strategies for diabetic kidney disease

**DOI:** 10.3389/fendo.2025.1513895

**Published:** 2025-06-11

**Authors:** Lin Wang, Shaowei Ding, Yuxin Hu, Jiaming Su, Gegongming Zhu, Hanzhang Hong, Baoluo Hou, Zhaoxi Dong, Zeyu Xue, Jiayi Wang, Zhongjie Liu, Hongfang Liu, Weijing Liu

**Affiliations:** ^1^ Dongzhimen Hospital, Beijing University of Chinese Medicine, Beijing, China; ^2^ Renal Research Institution of Beijing University of Chinese Medicine, Key Laboratory of Chinese Internal Medicine of Ministry of Education and Beijing, Dongzhimen Hospital Affiliated to Beijing University of Chinese Medicine, Beijing, China; ^3^ Beijing University of Chinese Medicine, Beijing, China; ^4^ Nephrology Department, Dongzhimen Hospital, Beijing University of Chinese Medicine, Beijing, China

**Keywords:** diabetic kidney disease, programmed cell death, apoptosis, pyroptosis, ferroptosis, autophagy

## Abstract

Diabetic kidney disease (DKD) is a leading cause of kidney failure. However, its pathogenesis remains incompletely understood, hindering the development of effective treatments. In recent years, substantial evidence has indicated that abnormal programmed cell death (PCD), including apoptosis, pyroptosis, ferroptosis, and autophagy, plays a crucial role in the progression of DKD, particularly in intrinsic renal cells such as podocytes, tubular epithelial cells, and mesangial cells. Novel therapeutic agents, such as sodium-glucose cotransporter 2 (SGLT2) inhibitors, glucagon-like peptide-1 (GLP1) receptor agonists, dipeptidyl peptidase-4 (DPP4) inhibitors, and relevant traditional Chinese medicines and their formulations, have demonstrated significant efficacy in improving intrinsic renal cell PCD in DKD. This review aims to provide a concise overview of the four types of PCD and their relationship with DKD, with a particular focus on highlighting the therapeutic potential of targeting PCD signaling pathways in the treatment of DKD.

## 1 Introduction

DKD is one of the most significant and severe microvascular complications of diabetes. DKD is characterized by persistent increase in urinary excretion of albumin, along with progressive reduction in glomerular filtration rate (GFR), ultimately leading to kidney failure (1). Approximately 20% to 40% of individuals with diabetes worldwide develop DKD (2, 3), and it is a major cause of kidney failure (4). Despite existing treatment strategies that incorporate lifestyle modification and pharmacotherapy, the risk of DKD and its associated complications remains high. DKD develops through distinct stages. The initial stage involves renal enlargement with increased GFR and perfusion, without significant renal pathological changes. Then pathological alterations become evident, with a decline or maintenance of estimated GFR and persistent microalbuminuria. Subsequently, there occurs a thickening of the glomerular basement membrane (GBM), an increase in the size of the glomeruli, and scarring resulting from high pressure within the glomeruli. Additionally, there is an expansion of the mesangial cells due to the accumulation of extracellular matrix (ECM), leading to a decline in renal function (1). As a systemic metabolic syndrome, the development and progressions of DKD involve multiple factors including metabolism, hemodynamic, lipid metabolism abnormalities, chronic inflammation, and accumulation of reactive oxygen species (ROS), which can lead to dysregulation of signaling cascades and intrinsic renal cell damage including podocytes, tubular epithelial cells, endothelial cells, mesangial cells, and other cell types (5). However, the exact mechanisms underlying the intrinsic renal cell damage have not been fully elucidated, and clinical intervention options remain limited.

To maintain the kidney function and homeostasis, a delicate balance between renal cell death and regeneration exists in human body. Cell death occurs during processes such as growth, metabolism, immune regulation, and hypoxic injury. The clearance of dysfunctional, infected, or potentially mutated cells in the body is driven by PCD pathways, which is key to the maintenance of internal environment stability, the defense mechanisms against pathogens, and a range of other physiological and pathological processes (6). PCD includes several well-described types, such as apoptosis, pyroptosis, ferroptosis, and autophagy, each employing different molecular and cellular processes with distinct outcomes (7). Apoptosis is a regulated, non-inflammatory form of cell death. Pyroptosis is an inflammatory form of cell death. Ferroptosis is primarily characterized by the accumulation of iron ions. Autophagy is a self-degradative cell death process that is essential for maintaining survival. Recent research has revealed the involvement of PCD of various intrinsic renal cells in the occurrence and progression of DKD. It can be illustrated by the case of podocyte apoptosis, as PCD plays a significant role in reducing the number of podocytes and causing glomerular injury. These effects are closely linked to proteinuria and glomerular damage in DKD. Similarly, the apoptosis of epithelial cells in the proximal tubules can lead to tubular atrophy, resulting in the loss of tubular cells and the development of tubulointerstitial fibrosis, ultimately leading to a decline in renal function. Additionally, decreased or impaired autophagy activity is also associated with the development of DKD. Reduced autophagy in podocytes impairs their function, disrupting the glomerular filtration barrier. Accumulation of damaged macromolecules and organelles in the proximal convoluted tubules occurs when autophagy of epithelial cells is reduced here, causing significant proteinuria and promoting the progression of DKD. Therefore, the pathological and physiological processes of DKD will be further elucidated for accurate understanding of the mechanism of PCD. Targeting and modulating specific PCD pathways and their corresponding signaling pathways may hold promise for mitigating renal damage and improving DKD.

In this review, we provide a comprehensive overview of the latest advancements in PCD research, and summarize the recent progress regarding the potential roles of PCD in the progression of DKD. Specifically, we focus on apoptosis, pyroptosis, ferroptosis, and autophagy in the context of DKD. To facilitate readability, we have listed all abbreviations in **Supplementary Table 1**.

## 2 Apoptosis

### 2.1 Characteristics and major signaling pathways

**Figure 1:**
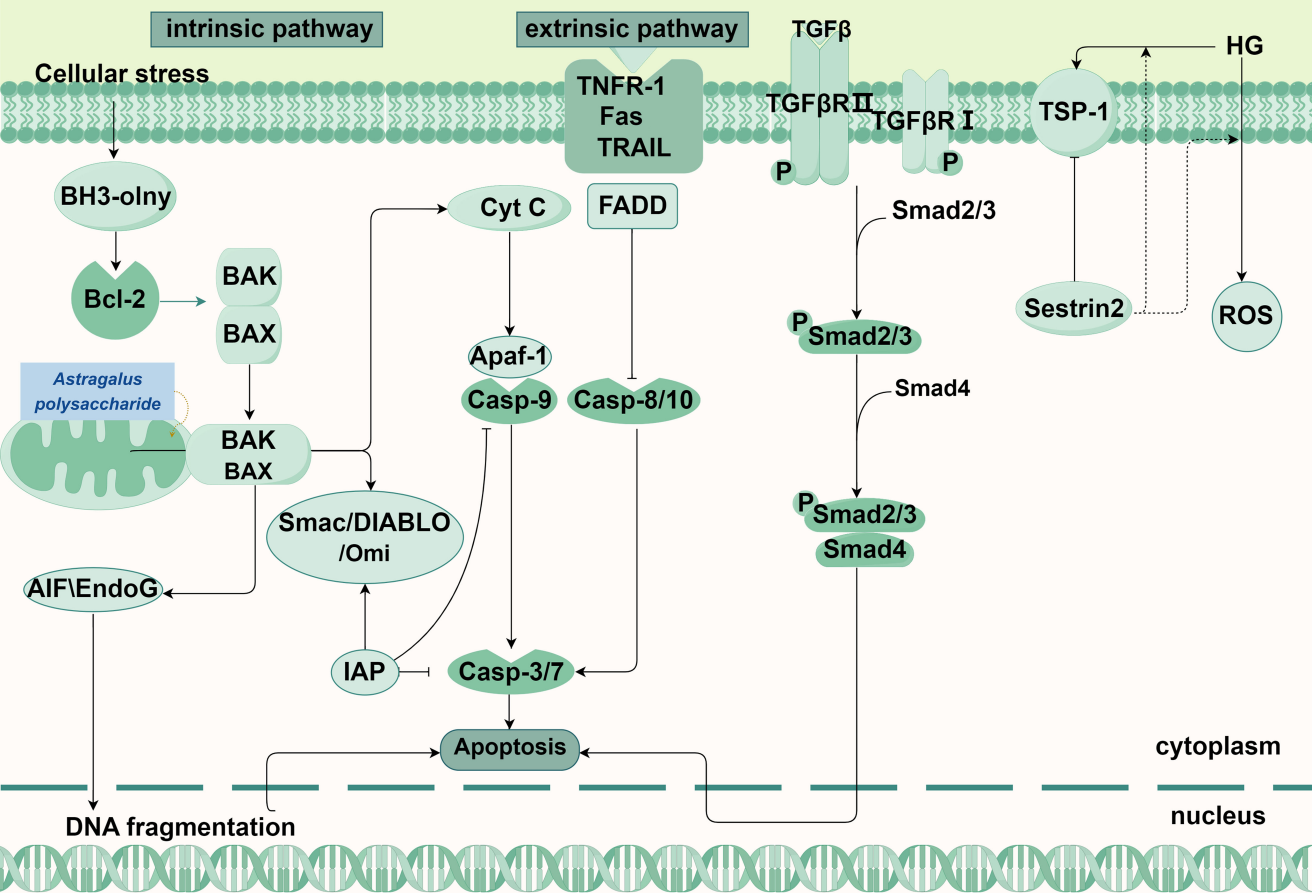
Main pathways in apoptosis and its relationship with DKD.

Apoptosis is a PCD process mediated by the caspase family, characterized by cytoplasmic shrinkage, chromatin condensation, nuclear fragmentation, cell membrane blebbing, and the formation of apoptotic bodies, as well as certain biochemical changes such as the exposure of phosphatidylserine to the outer plasma membrane (8–10). Apoptosis eventually leads to nuclear membrane disruption, the cleavage of many intracellular proteins (e.g., polymerase and layer membrane protein), membrane foaming, and the breakdown of genomic DNA into nucleosomal structures. Two main pathways exist in apoptosis: the intrinsic pathway and the extrinsic pathway (**Figure 1**) (11). Besides, Studies have found that many pathways can directly or indirectly cause apoptosis, such as TGF-β/Smad signaling pathway (12).

Initiated by non-receptor-mediated and characterized by mitochondrial regulation, the intrinsic pathway occurs mainly in the cell (13). Cellular stress can trigger an imbalance in the B-cell lymphoma 2 (Bcl-2) family members, resulting in the loss of mitochondrial membrane potential and an increase in permeability, leading to the formation of the mitochondrial permeability transition pore. This allows pro-apoptotic factors to be released into the cytoplasmic matrix from the mitochondria (14). Cytochrome C released from the mitochondria binds to apoptotic protease-activating factor 1 (Apaf-1) in the presence of deoxyadenosine triphosphate or adenosine triphosphate (ATP), forming the apoptosome complex (15, 16). Apoptosome complex recruit and activate Caspase-9, leading to the formation of apoptotic bodies and further activation of Caspase-3 and Caspase-7 (17, 18). In addition, the caspase activator from mitochondria is also released into the cytoplasmic matrix, binds to the apoptosis-inhibiting protein and inactivates it, thereby enabling apoptosis to proceed (19).In addition, there are other apoptotic factors in the Caspase-independent pathway, such as apoptosis-inducing factors (AIFs), endonuclease G and Caspase-Activated DNase (CAD), which are released from the mitochondria into the cytoplasm, leading to the occurrence of apoptosis (20).

The extrinsic pathway involves the activation of apoptosis through the binding of extracellular ligands to the transmembrane domains of death receptors (9). The extracellular ligands encompass tumor necrosis factor (TNF) and TNF-related apoptosis-inducing ligand (TRAIL). Receptors include type 1 TNF receptor (TNFR1), Fas, DR3, TRAIL-R1 (DR4), TRAIL-R2 (DR5), etc. These ligands selectively interact with distinct receptors, with TNF binding to TNFR1 and TRAIL binding to its specific TRAIL receptor (21, 22). After that, it recruits and binds the cytoplasmic adaptor protein (Fas-associated protein with death domain, FADD) and procaspase-8/-10 to form a death-inducing signaling complex (DISC). In DISC, procaspase-8/-10 autocatalytically cleaves into active caspase-8, followed by caspase-3 activation, resulting in the cleavage of various proteins within cells (20).

The TGF-β/Smad signaling pathway primarily consists of TGF-β and its receptors, as well as various Smad proteins (23). After being recruited to the cell by its type III receptor from the ECM to the vicinity of the cell, active TGF-β further binds to its type II receptor, which then activates type I receptors and downstream effectors Smad2 and Smad 3. At this, activated Smad2 and Smad 3 form a complex with Smad 4, activated smad complex is transported to the nucleus, thus regulating target genes, leading to the occurrence of apoptosis (24, 25). In the latest study, Kobayashi H et al. found that circulating NBL1 promotes apoptosis of kidney cells, especially podocytes. They predicted that inhibition of NBL1 to release BMP proteins can enable their interaction with and inhibition of TGF-β signaling (26) (**Figure 1**).

There are two routes of apoptosis, either intrinsic and extrinsic. On the left is the intrinsic pathway. Intracellular stress stimulates BH3-only protein and promotes the attachment of BAK or BAX to the outer mitochondrial membrane to form a mitochondrial permeability transition pore, leading to the release of apoptotic factors into the cytoplasm, where cytochrome C binds to Apaf-1 and the formed apoptosome complex recruits and activates Casp-9 to further activate Casp-3/7. Besides, EndoG and AIF in the mitochondria are released and move into the nucleus to break the DNA. TCM interventions: Astragalus polysaccharide (Astragalus membranaceus): Maintains mitochondrial function and suppresses apoptosis via AMPK/SIRT1/PGC-1α. In the middle is the extrinsic pathway. The combination of extracellular ligand and death receptors, successively recruits FADD, Casp-8/10, After self-activation of Casp-8/10, Casp-3 is further activated, leading to cell apoptosis, On the right are the pathways that can cause apoptosis in the DKD. Active TGFβ can further binds to TGFβR II recruited by the type TGFβRIII receptor, which then activates TGFβRI and the downstream effector Smad 2, Smad 3, phosphorylated Smad2 and Smad 3, and then transfer to the nucleus to regulate DNA transcription, leading to apoptosis.

Abbreviations: BH3-only: bcl-2 homology domain only proteins, Bcl-2: B-cell lymphoma-2, BAK: Bcl-2 homologous antagonist/killer, BAX: Recombinant Bcl2 Associated X Protein, Cyt C: Cytochrome Complex C, Casp: cysteine aspartate protease, Apaf-1: apoptotic protease activating factor-1, IAP: Inhibitor of apoptosis proteins, Smac: second mitochondria-derived activator of caspases, AIF:apoptosis inducing factor, EndoG: Endonuclease G, Fas: factor related Apoptosis, FADD: Fas Associating Death Domain Containing Protein, TNFR-1:Tumor Necrosis Factor Receptor 1, TRAIL: Tumor Necrosis Factor Related Apoptosis Inducing Ligand, TβRI/II/III: Recombinant Transforming Growth Factor Beta Receptor I/II/III, TGF-β: transforming growth factor β, TSP: thrombospondin.

### 2.2 Apoptosis in DKD

Apoptosis plays an important driving role in the progression of DKD. In patients with DKD and animal models, TGF-β ligands, TGFBRs (TGF-β receptors, and downstream signaling molecules such as Smad2 and Smad3 are highly upregulated or activated in the glomerulus, tubules, and renal interstitium (27, 28). Schiffer et al. found that the expression of pro-apoptotic protein Smad1 was positively correlated with early glomerulosclerosis in a TGF-β7 transgenic mouse model (29). Targeting the TGF-β/Smad signaling pathway could reduce apoptosis in glomeruli, tubules and renal interstitium (30).

Kobayashi H et al. found that circulating NBL1 leads to early kidney structural lesions through apoptosis of kidney cells, especially podocytes, which in turn may contribute to progressive kidney function decline and progression to ESKD. They predicted that inhibition of NBL1 to release BMP proteins can enable their interaction with and inhibition of TGF-β signaling, therefore diminishing inflammation and fibrosis in kidney tissue and podocyte loss in DKD. This protein may be an accessible target to slow DKD progression to ESKD. It may be a more appropriate target in this pathway than TGF-β directly (26).

Under pathological conditions of DKD, an excessive production of ROS by podocytes activates the antioxidant defense system, leading to the release of ROS, damage to cellular oxidative components, and ultimately causing apoptosis. However, we can protect podocytes from apoptosis by targeting cellular oxidative stress-related pathways (31). For example, overexpression of Sestrin2 reduces high glucose-induced TSP-1 and ROS generation, where TPS-1 also promotes ROS generation. Sestrin2 can interact with TSP-1 and inhibit the induction effect of TSP-1 on TGF-β1/Smad 3. Therefore, Sestrin2 can alleviate oxidative stress and alleviate the apoptosis and damage of podocytes in DKD by coordinating the downstream TSP-1/TGF-β1/Smad 3 pathway (32). Chen et al. discovered that p53 can be activated by retinoic acid receptor responder protein 1 on podocytes, leading to podocyte apoptosis (33). Xu et al. found that SPAG5-AS1 inhibits podocyte autophagy and exacerbates podocyte apoptosis through the SPAG5/AKT/mTOR pathway (34). In addition to podocytes, tubular epithelial cells are found to be particularly susceptible to the dysregulation caused by diabetic conditions due to their high energy demands and dependence on aerobic metabolism. The occurrence of renal cell apoptosis gives rise to two hallmark features of advanced DKD: tubular atrophy and a significant reduction in the population of tubular epithelial cells (35). Tubular epithelial cells and macrophages form a feedback loop through extracellular vesicle transfer, promoting renal inflammation and renal cell apoptosis in DKD (36).

## 3 Pyroptosis

### 3.1 Characteristics and major signaling pathways

Pyroptosis is a newly discovered form of PCD that is associated with inflammatory responses. It is characterized by cell swelling and cell membrane rupture leading to the release of cellular contents, and activation of intense inflammatory responses (37). Pyroptosis, being the most immunogenic type of cell death, exhibits a faster progression and triggers the release of a multitude of pro-inflammatory cytokines when compared to apoptosis (38, 39).

**Figure 2:**
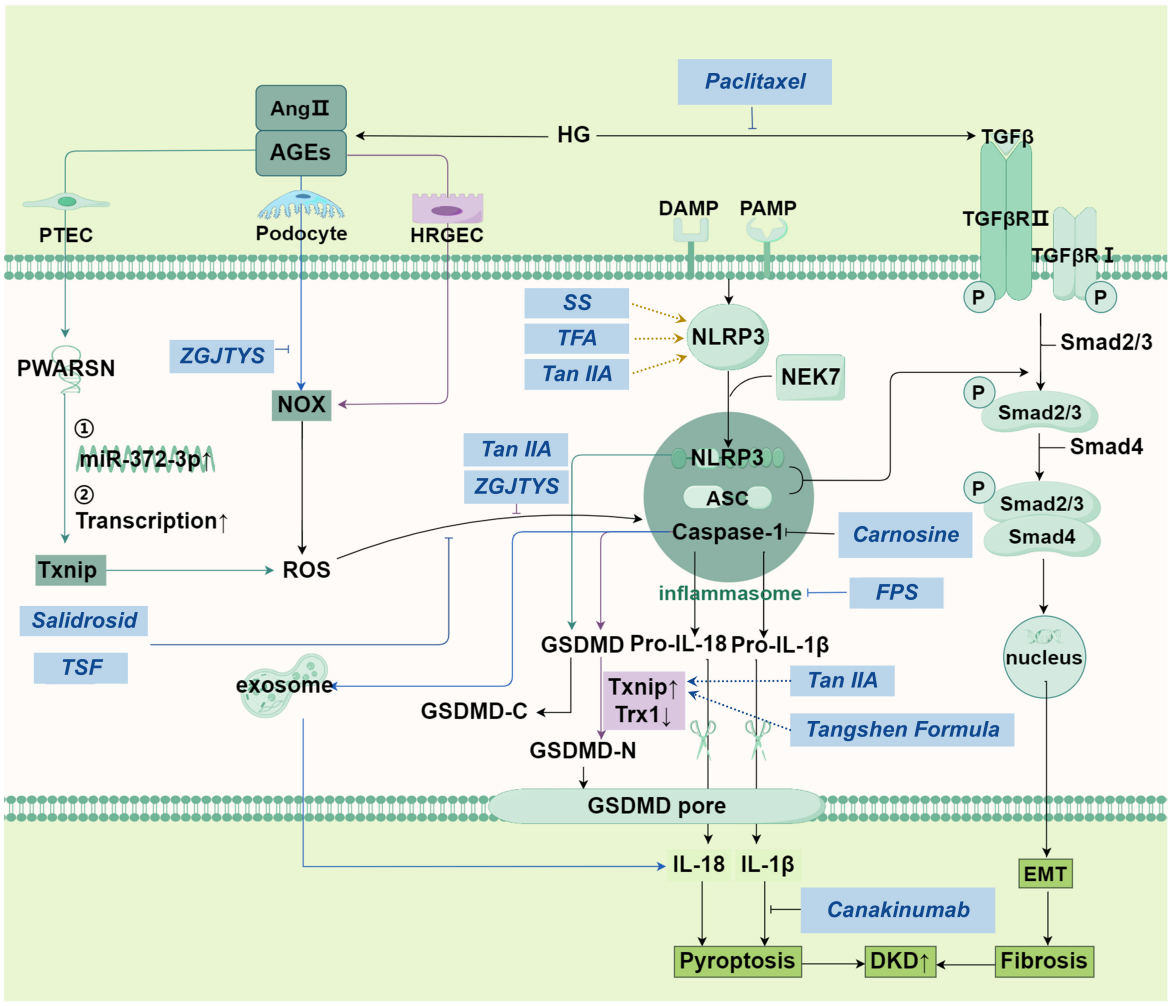
Main pathways in pyroptosis and its relationship with DKD.

The classical pathway of pyroptosis is initiated through the assembly of inflammasomes, which are complex structures formed by NLRP3, ASC (apoptosis-associated speck-like protein containing a caspase recruitment domain, and pro-Caspase-1 (**Figure 2**) (40). Upon stimulation by intrinsic or extrinsic stress signals, pattern recognition receptors are stimulated, and then NLRP3 binds to NEK7 to trigger its oligomerization (41), then pro-Caspase-1 is activated, which further cleaves pro-IL-18 and pro-IL-1β into mature IL-18 and IL-1β (42). Additionally, the gasdermin family of proteins, which possess pore-forming activity in immune responses, is activated by caspase-1 (42). Activated gasdermin family of proteins release the N-terminal domain that perforates the cell membrane, resulting in cell membrane rupture and the release of inflammatory factors such as IL-6 and TNF-α (43–45).

The non-classical pyroptosis pathway directly activates the caspase proteins, such as caspase-4, caspase-5 and caspase-11, through other pattern recognition receptors such as LPS, to mediate pyroptosis. Taking the non-classical communication pathway of GSDMD as an example, the activation of caspase leads to the cleavage of GSDMD, which triggers the efflux of K+. K+ efflux in turn activates the assembly of NLRP3 inflammasomes, leading to the cleavage of pro-IL-1β and pro-IL-18 (47). This triggers a cytokine storm, causing pyroptosis (**Figure 2**).

In DKD, podocytes are the important target cells of pyroptosis and have specific manifestations. NLRP3 gain -of-function mutation in podocytes, such as Nlrp3 Gate-of-function mutant (NLRP3A350V), significantly aggravated glomerular damage (such as albuminuria and mesangial dilatation) of DKD. Podocyte specific knockout of NLRP3 or caspase-1 protects kidney function completely or partially (46).

The left of the figure represents the mechanism of pyroptosis in PTEC, podocyte and HRGEC cells (colored in purple) respectively; In the middle is the classical pathway of pyroptosis. Under danger signal stimulation, an inflammasome is formed, which activates pro-Caspase-1 and converts it into active Caspase-1. Caspase-1 further cleaves pro-IL-18 and pro-IL-1β, generating mature IL-18 and IL-1β. Gasdermin D is activated by active Caspase-1, releasing its N-terminal domain. This domain perforates the cell membrane, leading to membrane rupture and the release of a large amount of inflammatory factors (such as IL-6 and TNF-α), triggering cell pyroptosis. The mechanism shown on the far right is similar to apoptosis: TGF-β is transferred to the nucleus through serial reactions to regulate DNA transcription and finally, lead to pyroptosis. SS alleviate HG-induced pyroptosis and oxidative damage by regulating the Nrf2/NLRP3 signaling pathway. Tan IIA: Inhibits ROS-Txnip/NLRP3 axis, mitigating oxidative stress-induced pyroptosis. Carnosine: Targets Caspase-1 to block podocyte pyroptosis, alleviating albuminuria and renal damage. Zuogui-Jiangtang-Yishen Decoction: Reduces podocyte pyroptosis by suppressing intestinal-derived TMAO via mROS-NLRP3 axis. TSF: Inhibits NLRP3 activation via ROS-Txnip axis, attenuating renal injury. FPS suppresses podocyte pyroptosis mediated by the NLRP3 inflammasome by regulating the AMPK mTORC3/NLRP1 signaling axis in DKD. Abbreviations: Ang II: angiotensin II, AGEs: Advanced glycation end-products, HG: high glucose, PTEC: Proximal tubular epithelial cells, HRGEC: human renal glomerular endothelial cell, NOX: NADPH Oxidase, NLRP3: The NACHT, LRR, and PYD domains-containing protein 3, NEK7: NIMA-related kinase 7, ASC: adapter protein apoptosis associated speck-like protein containing a CARD, IL-18: interleukin-18, EMT: epithelial-mesenchymal transition, ROS: reactive oxygen species, PWARSN: Prader Willi/Angelman region RNA, SNRPN neighbor, SS: sorasone base, Tan IIA: Tanshinone IIA, ZGJTYS: Zuogui-Jiangtang-Yishen Decoction, TSF: Tangshen Formula, FPS: Fucinose gum.

### 3.2 Pyroptosis in DKD

#### 3.2.1 Lead to ECM deposition and renal interstitial fibrosis

Previous studies have found that in a streptozotocin-induced diabetic mouse model, following an increase in blood glucose and creatinine levels, there is deposition of renal glomerular matrix and increased collagen fiber proliferation in the kidney tissue (47). Some renal tubules show cell swelling, vacuolar degeneration, tubular dilation, and thickening of the basement membrane. Additionally, it has been observed that the expression of GSDMD and Caspase-1 significantly increases in the kidney tissue of DKD mice, confirming the occurrence of pyroptosis mediated by Caspase-1 activation and triggered by GSDMD in the kidney tissue of DKD mice (47). During the process of pyroptosis, the aggregation of NLRP3 and ASC in renal intrinsic cells have been found to play a regulatory role in TGF-β/Smad-mediated ECM signaling. NLRP3 and ASC are essential for the phosphorylation of Smad2 and Smad3 (47).

In diabetic conditions, hyperglycemia and its metabolites stimulate TGF-β1 expression. Activated TGF-β binds to the type 2 transmembrane receptor (TGFBR2), which recruits and activates Smad2 and Smad3 regulated by TGFBR1 phosphorylated receptors through phosphorylation. Activated R-Smads can form complexes with Smad4 (common Smad or Co-Smad) and translocate into the nucleus to regulate the transcription of fibrosis genes (48).

NLRP3 enhances TGF-β signal transduction by promoting TGF-β1-induced Smad3 phosphorylation during EMT (47). Therefore, NLRP3 deficiency attenuates TGF-β1-triggered EMT and renal fibrosis *in vitro* and in diabetic mouse models (49).

Knockout of NLRP3 in mouse renal tubular epithelial cells (RTECs) has been shown to significantly downregulate the expression of TGF-β1 and its downstream targets, including EMT, MMP-9, and α-SMA (47). Furthermore, it has been found that inhibiting the activity of NLRP3 or inhibiting the activation of Caspase-1 can significantly suppress the overexpression of ECM proteins such as fibronectin and collagen induced by TGF-β1 in the kidney tissue of streptozotocin-induced diabetic mice and in high-glucose-induced mouse mesangial cells, thereby protecting against renal fibrosis (50).

#### 3.2.2 Oxidative stress caused by hyperglycemia causes pyroptosis in different renal cells

Hyperglycemia and various diabetes-related stimuli, including advanced glycation end-products (AGEs), AngII and TGF-β, are involved in increasing the expression and activity of various NADPH Oxidase (NOX) subtypes, thereby generating unwanted ROS in different cells and causing oxidative damage to renal tissue (51).

Podocyte injury and loss are important early pathological markers of DKD, which can accelerate the development of DKD (52). Studies have shown that pyroptosis is associated with podocyte loss (53). Hyperglycemia activates NOX in podocytes, thereby inducing the production of ROS, activating the specific NLRP3 inflammasome NLRP3A350V in podocytes, significantly promoting the secretion of inflammatory exosomes (MVB) in podocytes, releasing inflammatory cytokines such as IL-1β, and death in the GBM, thereby destroying the glomerular filtration barrier, aggravating kidney damage, and further developing DKD (46, 54, 55).

Human renal glomerular endothelial cells (HRGECs) are one of the structures in the glomerular filtration membrane and play an important role in charge and mechanical barrier in glomerular filtration. Their structure and dysfunction directly affect the glomerular filtration function and aggravate the occurrence and development of DKD. High glucose induces pyroptosis of glomerular microvascular endothelial cells by inducing the expression of ROS and superoxide anion in HRGECs. Thioredoxin-interacting protein (Txnip)-Trx oxidosome is an important regulator of redox signal transduction and an important contributor to the enzyme system related to ROS production and renal oxidative stress (56). Txnip can bind to the sulfhydryl group of thioredoxin 1 (Trx1) protein to form oxidase bodies, causing oxidative damage to cells (57). Previous studies have confirmed that oxidative stress is an important factor in the activation of NLRP3 inflammasome (58).

RTECs injury plays an important role in renal fibrosis. Recent studies have identified a new long-chain non-coding RNA-Prader Willi/Angelman region RNA, SNRPN neighbor (PWARSN), which is highly expressed in proximal tubular epithelial cells (PTECs) under high glucose conditions and regulates Txnip-induced PTEC pyroptosis in DKD (59). Studies have reported that hyperglycemia promotes the binding of Txnip and thioredoxin, leading to excessive oxidative stress and ROS production, then initiates DKD pyroptosis in a NLRP3 inflammasome-dependent manner (60, 61). PWARSN regulates Txnip expression in a dual way. On the one hand, it promotes the expression of Txnip protein through miR-372-3p to activate NLRP3 inflammasome (59). On the other hand, it promotes the transcription of Txnip gene by degrading nuclear RNA-binding motif protein X-linked protein (RBMX), increases the level of Txnip, promotes the activation of NLRP3 inflammasome and promotes pyroptosis (59).

It can be seen that under the stimulation of pathogenic factors in DKD, activation of the pyroptosis pathways can lead to increased ECM production, resulting in ECM deposition and renal interstitial fibrosis, exacerbating kidney damage in diabetic nephropathy. Therefore, antagonizing cell pyroptosis may serve as a promising strategy to alleviate renal interstitial fibrosis in diabetic nephropathy (62).

## 4 Ferroptosis

### 4.1 Characteristics and major signaling pathways

In 2012, Dixon first proposed the concept of iron death, a unique form of PCD characterized by dysregulation of iron-mediated lipid peroxide metabolism, leading to imbalance of the redox system and ultimately to oxidative damage to cell membranes and proteins (63–66).

**Figure 3:**
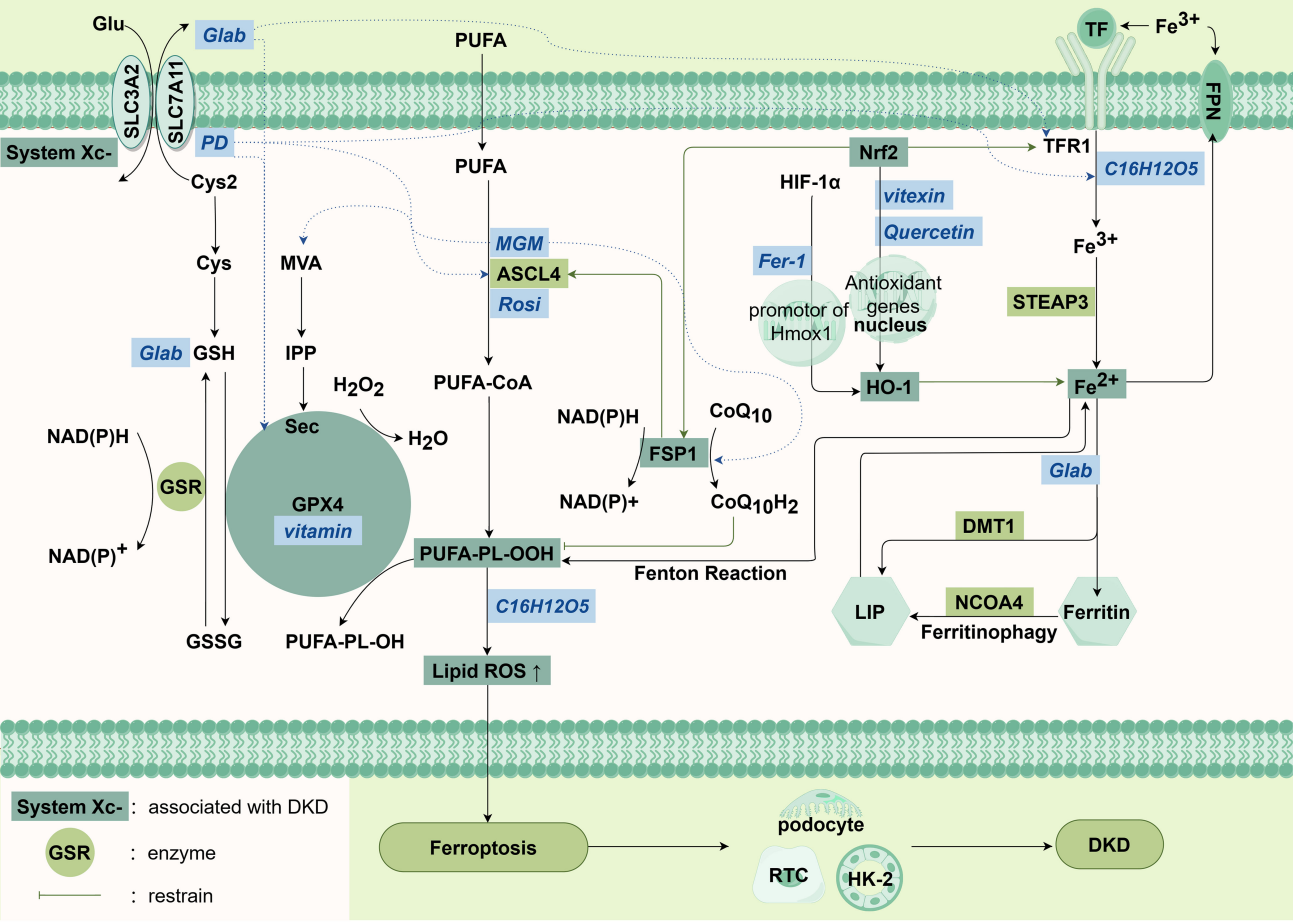
Main pathways in ferroptosis.

At present, the research on iron death focuses on three classical pathways: systemic Xc-/glutathione (GSH)/GPX 4 signaling pathway, iron metabolism signaling pathway, lipid metabolism pathway, and the emerging FSP 1/CoQ 10/NADPH system (65) (**Figure 3**).

System Xc− is an amino acid antiporter across the plasma membrane, facilitates the release of cystine (Cys2, an oxidized form of Cys) into the extracellular space, while simultaneously expelling glutamate (Glu) from the cell in a 1:1 ratio (67). Intracellularly, Cys2 is reduced to cysteine (Cys), and Cys undergoes an enzymatic reaction to ultimately produce GSH, an essential cofactor for glutathione peroxidases (GPXs). These GPXs play a crucial role in breaking down hydrogen peroxide and lipid peroxides, thereby inhibiting the formation of lipid ROS and suppressing ferroptosis (68, 69). The mevalonate (MVA) pathway can affect the synthesis of selenocysteine tRNA by down-regulating isopentenyl pyrophosphate (IPP), further interfere with the activity of GPX4, and cause ferroptosis (70).

Ferroptosis is closely associated with the body’s iron metabolism homeostasis. In normal physiological conditions, the cell membrane’s transferrin receptor 1 (TFR1) facilitates the entry of Fe3+ into cells by transporting the complex that formed with the binding of transferrin (TF) and Fe3+, where it is then converted to Fe2+ by the STEAP3 protein and finally stored in the cell (71). Most of the intracellular iron is stored in ferritin form, while a small portion is transported into the cytoplasmic labile iron pool (LIP) through divalent metal transporter 1 (DMT1) (72, 73). Ferroportin (FPN), a membrane iron transporter, oxidizes excess Fe2+ to Fe3+ and mediates the export of iron from cells. In situations of excessive iron accumulation, an abundance of free Fe2+ within the LIP can generate hydroxyl radicals via Fenton reaction. These radicals actively participate in lipid peroxidation reactions and trigger ferroptosis (74). On the other hand, excess Fe2+ induces the activation of iron enzymes such as lipoxygenases (LOXs), further promotes lipid peroxidation in the cell membrane, and ultimately leads to cell death (75).

The accumulation of polyunsaturated fatty acids (PUFAs) is widely recognized as a characteristic feature of ferroptosis (76). This process involves the buildup of fatty acids, including both polyunsaturated and monounsaturated forms (MUFAs), which serve as substrates for lipid peroxidation (77). PUFAs are more susceptible to oxidation compared to MUFAs. Consequently, increasing PUFA levels while reducing MUFA content can facilitate lipid peroxidation and contribute to the progression of ferroptosis. Additionally, crucial enzymes participating in these enzymatic processes encompass long-chain acyl-CoA synthetase 4 (ACSL4), lysophosphatidylcholine acyltransferase 3 (LPCAT3), and arachidonate 15-lipoxygenase (ALOX15). ACSL4 facilitates the esterification of free PUFAs by incorporating them into membrane phospholipids with assistance from LPCAT3. Subsequently, ALOX15 plays a role in initiating the peroxidation process on membrane phospholipids (78). Therefore, it can be inferred that the ACSL4/LPCAT3/ALOX15 pathway promotes ferroptosis through lipid peroxidation.

In recent years, there has also been emerging research on the pathways involved in ferroptosis. Type II NADH ferroptosis inhibitor 1 (FSP1) has also been found. As an alternative mechanism of ferroptosis inhibition, the FSP1/CoQ10/NADPH system has nothing to do with the intracellular expression levels of GSH, GPX4 and ACSL4, and can protect cells from ferroptosis induced by GPX4 inhibition. FSP1 is mainly distributed in lipid droplets and plasma membranes, and can inhibit lipid peroxidation and ferroptosis by reducing lipid free radicals (79). (**Figure 3**)

Solute carrier family 7 member 11 (SLC7A11) is an active subunit of the cystine/glutamate reverse transporter system Xc− (80). Reduced SLC7A11 expression induces reduced cystine uptake, leading to depletion of GSH. p53 is a key tumor suppressor gene that functions as a transcription factor (80). Recent studies have shown that mitochondrial single stranded DNA binding protein 1 (SSBP1) induces phosphorylation of p53 serine 15 (S15) by DNA-dependent protein kinase (DNA-PK) and promotes nuclear accumulation of p53. Inhibition of SLC7A11 expression in high-fructose exposed podocytes drives ferroptosis (81).

Recent studies have found that ferroptosis involves the death of RTECs and podocyte in DKD (82). Due to the high sensitivity of podocytes to ROS, excessive ROS can cause irreversible changes in podocyte structure and function, leading to the occurrence of DKD. RTECs are one of the most mitochondria-rich cell types, so they are more susceptible to oxidative stress damage, which is also part of the pathogenesis of DKD, suggesting that this may be part of the cause of DKD caused by ferroptosis (83, 84).

There are four main mechanisms, from left to right are System Xc-/GSH/GPX4 signaling pathway, lipid metabolism pathway, the FSP1/CoQ10/NADPH system and iron metabolism signaling pathway. The italic characters in the blue box in the figure are abbreviations of drugs.

Abbreviations: Cys: cystine, Cys2: Cystine, Glu: glutamate, GSH: glutathione, GSSG: glutathione disulfide, GPX4: glutathione peroxidase 4, PUFAs: polyunsaturated fatty acids, SLC3A2: solute carrier family 3 member 2, SLC7A11: solute carrier family 7 member 11, GSR: glutathione-disulfide reductase Gene, MVA: the mevalonate pathway, IPP: isopentenyl pyrophosphate, Sec: selenocysteine, PLOOH: phospholipid Hydroperoxides, FSP1: ferroptosis inhibitor 1, Nrf2: nuclear factor erythroid2-related factor 2, HIF-1α: hypoxia inducible factor-1α, TF: transferrin, TFR1: transferrin receptor protein 1, STEAP3: DMT1: divalent metal transporter 1, HO-1: heme oxygenase-1, NCOA4: nuclear receptor coactivator 4, LIP: labile iron pool, ROS: reactive oxygen species, FPN: ferroportin, PD: platycodon grandiflorum D, MGM: mangiferin monosodium salt, Rosi: rosiglitazone, Fer-1: ferritin-1.

### 4.2 Ferroptosis in DKD

Studies have shown that ferroptosis indicators are up-regulated in DKD patients, and the expression of system Xc- and Gpx4 mRNA is also reduced in renal biopsy samples of DKD patients. In DKD animal models, there is a notable increase in iron content in renal tissue, which positively correlates with blood creatinine and urinary protein levels, as well as renal iron protein levels (85, 86). Iron overload, decreased antioxidant capacity, ROS accumulation, and id peroxidation are characteristic changes associated with iron death in the kidneys of streptozotocin-induced DBA/2J diabetic mice (87). Feng et al. observed that iron death causes damage in renal tubules through the hypoxia-inducible factor-1α (HIF-1α)/heme oxygenase-1 (HO-1) pathway (88). Treatment with the selective iron death inhibitor Ferostatin-1 (Fer-1) inhibits the expression of HIF-1α and HO-1, thereby alleviating renal tubular iron overload, inhibiting ROS formation, reducing oxidative stress and lipid peroxidation, thereby exerting protective effect against renal tubular injury and fibrosis in diabetic mice. To summarize, mitochondrial abundance determined the metabolic characteristics and baseline ROS levels of cells, and affected the sensitivity to ferroptosis. Oxidative stress both triggers ferroptosis and accelerates the process of ferroptosis by causing an imbalance in the antioxidant system.

In recent years, the key role of ferroptosis in the occurrence and development of kidney disease has been paid more and more attention, which may become an emerging target for clinical treatment and related drug development (89).

## 5 Autophagy

### 5.1 Characteristics and major signaling pathways

Autophagy is a highly conserved catabolic process that serves to degrade damaged proteins and organelles, ensuring the preservation of cellular homeostasis. Autophagy involves lysosomal degradation and recycling of unnecessary cytoplasmic proteins and organelles, which is considered a self-protective response to stress (90). Under normal physiological conditions, cells maintain low baseline levels of autophagy, and autophagy levels are rapidly increased under factors such as ROS, nutrient and energy deprivation, ER stress, hypoxia, DNA damage, and immune signaling. Mammalian cells exhibit three distinct types of autophagy, classified based on the substrate to be degraded and the pathway through which substrates are transported to lysosomes: autophagy, chaperone-mediated autophagy, and autophagy (73, 91, 92). The most extensively characterized form of autophagy is autophagy (hereafter simply referred to as autophagy), which involves the sequestration of substrates within double-membrane structures called autophagosomes that originate from the endoplasmic reticulum, followed by their fusion with lysosomes for content degradation (93). Autophagy exhibits a notable capacity for degrading entire organelles and large protein aggregates, making it the predominant form of autophagy (94).

**Figure 4:**
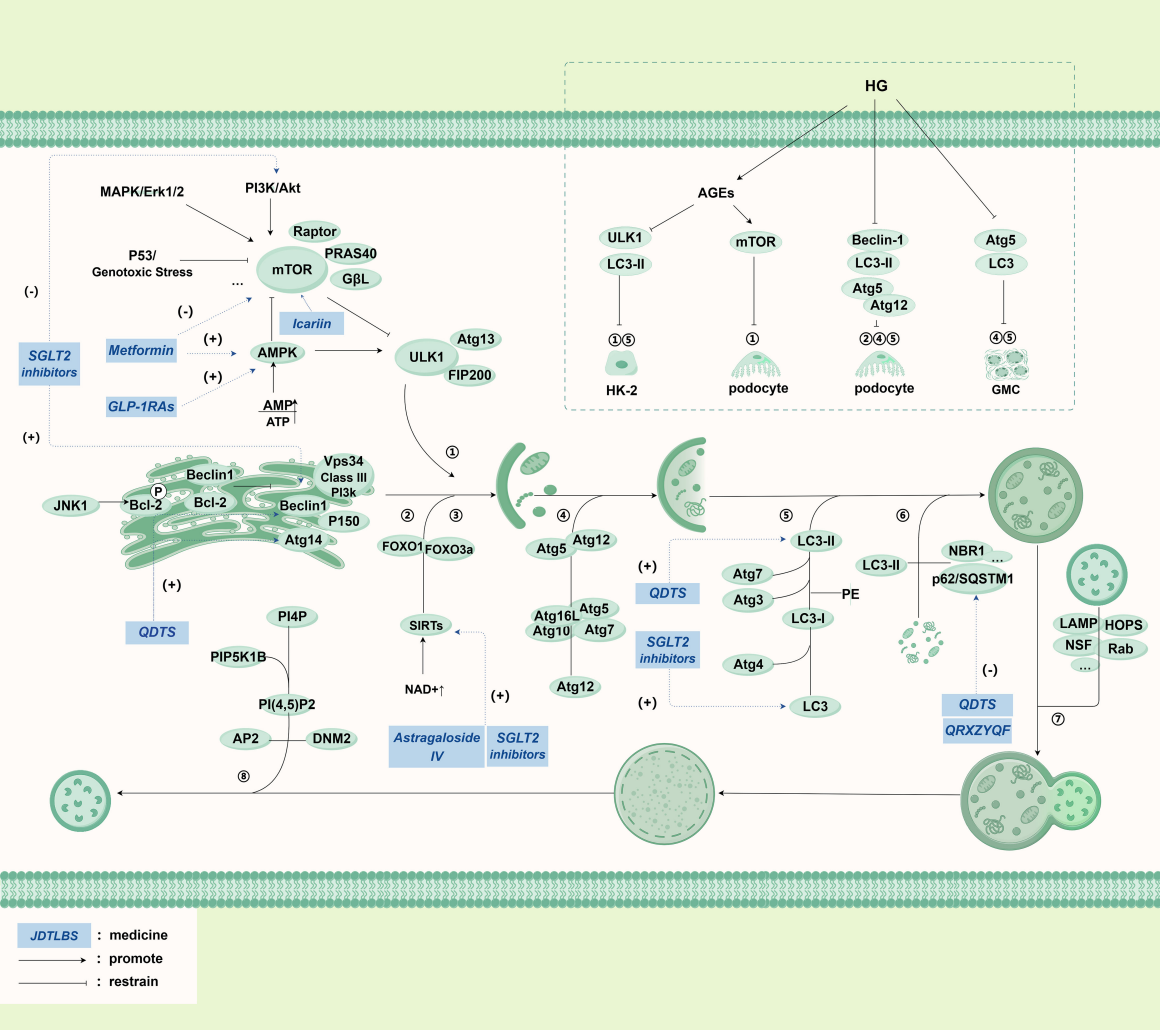
Main pathways in autophagy and its relationship with DKD.

Under normal physiological conditions, stress signaling kinases such as JNK-1 phosphorylate Bcl-2 to promote autophagy, which in turn affects regulatory proteins such as Beclin-1 to interact with class III phosphatidylinositol 3-kinase (PI3K) typical of Vps 34 to promote PI3P generation to extend with the phagocytotic membrane and attract Atg proteins. The onset of autophagy is influenced by multiple factors. The AMP-activated protein kinase (AMPK) regulates autophagy through a mammalian target of rapamycin (mTOR) -dependent and mTOR-independent pathways (95). In response to energy deprivation, the AMP/ATP ratio increases, promoting mTORC1 inactivation and ULK 1 complex activation, thereby increasing autophagy levels (96–98); AMPK can also directly initiate autophagy by phosphorylated ULK 1 (99), where activated mTORC1-phosphorylated ULK 1 disrupts the interaction with AMPK and suppresses autophagy. As mTOR integrates growth factors and nutrient signals that regulate cell growth and is responsible for inhibiting autophagy, mTOR is activated and phosphorylates specific sites on ULK 1 and ATG 13 to inhibit the activity of ULK 1-ULK 2 complex and inhibit the occurrence of autophagy (95, 100, 101), and is inhibited during starvation and cellular stress, leading to its dissociation from ULK 1 (102). ULK 1 phosphorylation promotes the recruitment and phosphorylation of various proteins, assembling the ULK 1 complex and translocates to the isolation membrane of the endoplasmic reticulum to initiate autophagy (100, 103). Moreover, Sirtuins (SIRTs) form a class of NAD + -dependent class III histone deacetylases actively involved in metabolic regulation. During cellular energy expenditure, NAD + levels increase, leading to the activation of downstream SIRT 1, and SIRT 1 directly deacetylates the transcription factor forkhead box proteins O1 (FOXO1) and FOXO3a to activate cellular autophagy and restore cellular energy homeostasis (104, 105). After initiation of autophagy, Atg 5-Atg 12 is processed under the participation of two ubiquitin-like coupling pathways, and LC3B-II integrated with it is embedded into the autophagosome membrane in the extension, becomes an autophagy marker and participates in membrane fusion and substrate selection. LC3B-II recognizes proteins such as p62/SQSTM1 on the target substrate, realizes autophagosome membrane closure after selective uptake, and realizes the fusion of lysosome with the help of related proteins such as SNAREs and Rab. After autolysosomes complete substrate degradation, the clathrin bud tube are formed, and the protolysosomes are formed by the breaking of the GTP enzyme DNM 2 (104) (**Figure 4**).

In addition to autophagy, the specific pathways and mechanisms underlying autophagy and CMA remain to be further elucidated. In autophagy, cytoplasmic components are actively and directly engulfed by the lysosomes through invagination of the lysosomal membrane, subsequently leading to degradation within the lysosomes (106). Chaperone-mediated autophagy relies on the specific recognition of cytoplasmic protein components containing the pentapeptide motif KFERQ by heat shock cognate protein 70 (Hsc70, also known as HSPA8), and the multimeric complex formed by lysosome-associated membrane protein 2 and other cofactors, facilitating the delivery of substrates to the lysosomes for degradation (107).

Podocytes exhibit characteristically high basal autophagy activity, requiring sustained autophagic flux to preserve their functional homeostasis. Autophagy regulation in these cells is principally mediated by AMPK signaling, with supplementary modulation through the JAK2-STAT1-Tfeb axis. While acute mTORC1 inhibition potentiates autophagy via AMPK activation, chronic inhibition results in re-establishment of baseline autophagic activity. Notably. In proximal tubular epithelial cells, physiological autophagy activity remains comparatively quiescent. Nevertheless, constitutive autophagy proves indispensable for cellular homeostasis and mitochondrial quality control, with particularly robust mitophagy observed in cortical segments to accommodate their substantial bioenergetic demands. Glomerular endothelial cells critically depend on autophagy-mediated ROS clearance to sustain capillary integrity. Autophagy impairment in these cells precipitates oxidative damage, manifesting as pathological glomerular capillary dilatation, mesangiolysis, and ultimately glomerulosclerosis (108).

The core molecular mechanism of autophagy is most extensively studied in autophagy, which is often referred to as “autophagy”. The main mechanism pathway of autophagy is presented in detail in the diagram, including phagophore formation, substrate uptake, autolysosome formation, functional lysosome regeneration and regulation pathway with mammalian target of rapamycin (mTOR) as the core. At the same time, the upper right part of the diagram introduces the mechanism of autophagy process in RTECs (HK-2), glomerular mesangial cells (GMC), podocytes and renal cortical epithelial cells under high glucose (HG) environment. The italicized words in the blue box represent drugs.

Abbreviations: JNK-1: c-Jun N-terminal kinase, Bcl-2: B-cell lymphoma-2, Vps34: Vacuolar Protein Sorting 34, PI3K: Phosphoinositide 3-Kinase, Atg: Autophagy related gene, FOXO1: Forkhead box O1, FOXO3a: Forkhead box O3a, SIRTs: Sirtuins, NAD+: Nicotinamide adenine dinucleotide, LC3: MAP1LC3, microtubule-associated proteins light chain 3, PE: Phosphatidyl Ethanolamine, p62/SQSTM1: Sequestosome 1, PI4P: Phosphatydyinositol 4-phosphate, PIP5K1B: Phosphatidylinositol-4-phosphate 5-kinase, type I, PI (4, 5)P2: Phosphatidylinositol-4, 5-bisphosphate, AP2: Adaptor Protein2, DNM2: Dynamin2, mTOR: Mammalian target of rapamycin, ULK1: unc-51 like kinase 1 Gene, AMPK: Adenosine 5’-monophosphate (AMP)-activated protein kinase, SGLT2 inhibitors: Sodium-glucose co-transporter 2 inhibitors, GLP-1RAs: Glucagon like peptide-1 receptor agonists, PF: Paeoniflorin, TG: Tripterygium Glycosides, QDTS: Qi Di Tang Shen granules, QRXZYQF: Qingre Xiazheng Yiqi Fang, JTBF: Jiedu Tongluo Baoshen formula.

### 5.2 Autophagy in DKD

During the progression of DKD, the autophagic activity of podocytes decreases, along with the reduced expression levels of autophagy-related proteins, including Beclin-1, LC3-II, and Atg5-Atg12 complex (109–113). In a diabetic mouse model, podocytes exhibited defective autophagy, decreased LC3-II levels, and accumulation of p62, indicating that high glucose reduces podocyte autophagic activity (114). In diabetic mice, the expression of AGEs is increased, which activates mTOR and inhibits the nuclear translocation of pro-autophagy transcription factor EB (TFEB) to inhibit the formation and turnover of podocyte autophagosomes (115). However, other studies have yielded inconsistent results. Another study found that during high glucose treatment, podocytes show increased expression of autophagy-related proteins LC3-II, Beclin-1, and autophagosomes (116, 117), suggesting that high glucose can promote podocyte autophagy. Similar results have also been observed in primary podocytes and podocyte cell lines (115, 118). We analyzed the impact of high glucose on SVI cells (a stable podocyte cell line) and found that 48 hours of high glucose treatment induced autophagy in SVI cells, confirming the short-term high glucose can induce podocyte autophagy. On the other hand, when longer-term exposure (15 days) of high glucose exhibit inhibitory effect on podocyte autophagy, as demonstrated by the decreased LC3B-II expression in SVI podocytes after a 15-day exposure to high glucose (119).

Compared to podocytes, RTECs display lower basal levels of autophagy (120). Both *in vitro* and *in vivo* studies have observed mitochondrial fragmentation and depolarization accompanied by autophagy inhibition in RTECs under high glucose conditions (121). This suggests autophagy system inhibition is correlated with mitochondrial dysfunction. Conversely, significant upregulation of LC3-II, autophagosomes, and Beclin1 levels were observed in HK-2 cells and kidneys of diabetic rats, suggesting possible activation of the autophagy pathway under high glucose conditions (122). However, it is also found that autophagy increases after 6 and 12 hours of high glucose culture in PTEC, but is suppressed after 48 or 96 hours. This further supports that short-term high glucose exposure may activate autophagy in response to cellular stress induced by high glucose, but prolonged exposure inhibits autophagy (117).

The autophagic activity of mesangial cells is the most controversial among all types of renal intrinsic cells. In a streptozotocin-induced diabetes model, stronger GFP-LC3 staining and conversion of LC3B-II in glomeruli were observed at 4 weeks, indicating increased autophagic activity in early diabetic glomeruli under high glucose induction. On the other hand, at 8 weeks, LC3 staining decreased and p62/SQSTM1 accumulated in the cytoplasm of glomerular cells, confirming the impairment of autophagic activity in glomeruli under long-term high glucose conditions (119). Specific depletion of Atg5 to inhibit autophagy significantly exacerbated the cytotoxic effects of advanced glycation end products in mesangial cells, suggesting that activated autophagy has a renal protective effect against advanced glycation end products-induced mesangial cell injury (123).

The above research findings suggest, we speculate that short-term exposure to high glucose induces cellular autophagy to protect the kidneys, but long-term high glucose exposure inhibits autophagy, resulting in aggravated damage to renal intrinsic cells and the progression of DKD.

## 6 PCD in DKD as a potential therapeutic target

### 6.1 Conventional treatment strategies

The management of DKD typically involves addressing lifestyle modification, glycemic control, blood pressure control, lipid management and reducing albuminuria. Metformin is commonly prescribed as the initial medication for regulating blood glucose in patients with T2DM. Emerging medications such as SGLT2 inhibitors, GLP1 receptor agonists (GLP-1RAs), non-steroidal mineralocorticoid receptor antagonists (nsMRAs) and renin-angiotensin system(RAS) inhibitors, which are known as the four pillars, offer additional treatment options for DKD (124, 125). Extensive research has demonstrated that these drugs possess renal protective properties and can effectively slow down the progression of DKD by modulating molecules and signaling pathways associated with PCD. However, nonspecific improvement of inflammation could be a secondary consequence of other beneficial effects, such as decreased glomerular hyperfiltration and albuminuria (126). Furthermore, it has been confirmed in retrospective cohort studies and clinical trials that DPP-4 inhibitors have no effect on renal outcomes except for albuminuria, so this kind of drugs will not be discussed here (127–130).

#### 6.1.1 Metformin

Metformin is one of the main medications used to treat diabetes, and research has shown that it can’t only lower blood sugar through various mechanisms but also prevent the progression of DKD to kidney failure (131). Previous research has indicated that metformin protects kidney function by promoting pathways mediated by AMPK, for instance not only it enhances the formation and subsequent removal of autophagic vesicles through signaling pathways mediated by AMPK and inhibition of mTOR (131), but also it can induce renal autophagy through the AMPK/SIRT1-FoxO1 pathway to alleviate kidney damage, improve oxidative stress, and correct glucose metabolism abnormalities (132). As a result, it provides protective effects for the kidneys in DKD by mitigating podocyte loss, apoptosis in mesangial cells, and senescence in tubular cells (133).

#### 6.1.2 SGLT2 inhibitors

SGLT2 inhibitors are a novel class of antidiabetic drugs. The ADA 2022 Standards Care and the KDIGO 2022 guidelines both recommend SGLT2 inhibitors as first-line drug therapy for patients with CKD and diabetes (134). These drugs promote uric acid excretion, lower blood uric acid levels, inhibit the reabsorption of sodium and glucose in the proximal tubules, and, in addition, stimulate afferent arteriolar vasoconstriction in the glomerulus by a volume-regulatory mechanism in the juxtaglomerular apparatus. This helps reducing glomerular hyperfiltration and thereby provides kidney protection (135). Furthermore, some studies have reported the mechanisms through which SGLT2 inhibitors regulate PCD for kidney protection in DKD.SIRT1/AMPK were activated by SGLT2 inhibitors, and Akt/mTOR signaling is inhibited at the same time, which regulate autophagy (136). In a DKD rat model, Dapagliflozin suppresses cell apoptosis and improves fibrosis by upregulating Bcl-2 and downregulating BAX, thereby inhibiting cell apoptosis (137). In another animal experiment, Dapagliflozin inhibits cell apoptosis by reducing the expression of pro-apoptotic gene (caspase-3) and increasing the expression of anti-apoptotic gene (Bcl-2 in the kidney. It also enhances autophagy by increasing the expression of LC-3 and Beclin-1 (**Figure 4**), thus reducing kidney fibrosis (138). Additionally, SGLT2 inhibitors can directly prevent the downregulation of the podocyte autophagic mechanism, increase podocyte autophagy levels, and improve podocyte cellular remodeling, thereby protecting podocytes (139). In mice models, low-dose empagliflozin improves renal fibrosis by modulating iron homeostasis and regulating the expression of genes related to oxidative stress (139) dapagliflozin effectively reduces serum ferritin levels and ameliorates disorders in iron metabolism among patients with type 2 diabetes mellitus (T2DM) (140). Experimental studies demonstrating that in kidney tissues of animals treated with SGLT2 inhibitors markers of inflammation and fibrosis, including NF-κB, CC-chemokine ligand 2 (CCL2), JNK–signal transducer and IL-6 decreased (141, 142). Recent study had found that SGLT2 inhibitors appear to reduce inflammatory markers such as TNF-1, IFNγ, TGF-βand to modulate autophagy, in part by activation of Nrf2/HO-1 pathways. Empagliflozin appears to reduce IL-β inflammatory pathway in proximal tubular cells and improve mitochondrial function (143–145).

#### 6.1.3 RAS inhibitors

It is recommended that patients with diabetes, hypertension, and albuminuria should be treated with an (renin-angiotensin system) RAS inhibitor, such as an angiotensin converting enzyme inhibitor (ACEI) or angiotensin II receptor blockers (ARB), at the highest tolerated dose (134). Several clinical trials have found that an RAS inhibitor decreased the risk of CKD progression (146, 147). As single agents, RAS inhibitors have been shown to slow the rate of progression of DKD and to reduce new cases of end-stage renal disease in various subsets of patients with diabetes and proteinuria (148). Ang2 induces oxidative stress via NADPH oxidase and promotes inflammation and fibrosis by activating cytokines such as NF-κB, monocyte chemoattractant protein-1 (MCP-1), and TGF-β. ACEi/ARBs counteract these pathogenic effects, with evidence confirming their efficacy in delaying diabetic nephropathy progression in both type 1 and 2 diabetes (149). However, due to its adverse risks, it has been basically abandoned. It had to be noticed that the combined use of an SGLT2 inhibitor and ACEI/ARB shows a prominent decline in renal function approximately by 30–40%, surpassing the effects observed with ACEI/ARB alone (150).

#### 6.1.4 GLP-1RAs

According to KDIGO, a GLP-1RA with proven cardiovascular benefit is recommended for patients with T2DM and CKD who do not meet their individualized glycemic target with metformin and/or an SGLT2i or who are unable to use these drugs (134). New evidence suggests that GLP-1RAs have kidney-protective effects independent of their glucose-lowering effects and play a role in inhibiting the occurrence and progression of DKD. These effects include preventing pancreatic beta-cell apoptosis, suppressing glucagon secretion, possessing antioxidative stress properties, and enhancing endothelial function (151). Studies suggest that GLP-1RAs may exert renal protective effects by positively regulating autophagic flux through the AMPK-mTOR signaling pathway (152). Semaglutide (Smg) shows renal protective properties in patients with DKD through ferroptosis inhibition across clinical subjects, animal models, and HK-2 cells. This ferroptosis suppression mediated by Smg subsequently reduces renal inflammatory responses and fibrotic progression through activation of the AMPK signaling pathway (153). Both liraglutide and Smg exert podocyte-protective actions via dual mechanisms involving NLRP3 inflammasome pathway modulation and pyroptosis suppression (154).

#### 6.1.5 nsMRAs

The nsMRAs, including finerenone, apararenone, esaxerenone, and ocedurenone, which distribute between heart and kidney tissue rather than influencing the kidney alone, are conspicuously different from steroidal-based MRAs which are restricted due to hyperkalemia. Data from both animal as well as human outcome studies encourage a possible additive effect of finerenone to reduce heart and kidney fibrosis as well as improve organ protection when used with SGLT2 inhibitors and possibly GLP-1RAs (155, 156).These drug not only slowed the progression of DKD, but also achieved remarkable efficacy, acting to reduce inflammation and fibrosis within the kidney (156). Preclinical studies has shown that nsMRAs reduce the upregulation of proinflammatory mediators, including TNF-α, IL-6, IL-1β, plasminogen activator inhibitor 1 (CXCL1), CCL2, and MCP-1 (157).

#### 6.1.6 Integrated management

In addition to lowering blood glucose, lowering blood pressure, lowering blood lipid and lifestyle intervention are also important components of DKD treatment. KDIGO 2022 guidelines suggests treating adults with hypertension and DKD to a target systolic blood pressure of <120 mmHg (134). RAS inhibitors are the preferred antihypertensive drug in DKD patients. RAS inhibitors is especially suitable for those with proteinuria. At present, the commonly used RAS inhibitors in clinical practice are mainly ACEi/ARB drugs such as captopril and irbesartan. However, no studies have reported that these drugs can target PCD to improve DKD. As dyslipidemia is a major cardiovascular risk factor in patients with type 2 diabetes, lipid-lowering agents are another important therapy for patients with DKD (158). The Study of Heart and Renal Protection trial aimed to assess the safety and efficacy of reducing low density lipoprotein (LDL) cholesterol in more than 9000 patients with CKD and showed that simvastatin 20 mg plus ezetimibe 10 mg daily safely reduced the incidence of major atherosclerotic events (159). However, there is also no report that lipid-lowering drugs can target PCD to treat DKD.

Lifestyle modification, including low salt diet and low protein diet, should be the initial intervention in the management of DKD (152). A low-salt and moderate low-protein diet can undoubtedly help control blood pressure and reduce proteinuria, but the role of PCD in it is still unknown. Loh JT et al. found that high-salt diet enhanced the resistance of bacteria to oxidative stress by inducing fur-R88H mutation of Helicobacter pylori, and promoted the expression of iron uptake-related proteins (such as siderophores) (160). Iron metabolism disorder and oxidative stress are the core mechanisms of ferroptosis, but this study did not directly verify the ferroptosis pathway. Di Castro S et al. found that high-salt diet down-regulated renal mitochondrial uncoupling protein 2, and UCP2 deficiency can lead to mitochondrial dysfunction and oxidative stress, which may indirectly affect the autophagy-lysosomal pathway (161). Zeng L et al. found that high-protein almond supplementation can increase renal GSH level and reduce oxidative stress, and GSH depletion is an important cause of ferroptosis, suggesting that high-protein diet may inhibit ferroptosis by maintaining GSH homeostasis, but the specific mechanism still needs to be verified (162). In addition, it has been reported that a high-salt diet increases oxidative stress in the kidney, suggesting that this oxidative stress may damage the kidney through an apoptotic pathway (161, 163). In conclusion, there is no direct evidence that low-salt and low-protein diets can treat DKD by targeting PCD.

Since quitting smoking, reducing alcohol consumption, and maintaining a low-fat diet are important for DKD treatment, we examined whether smoking, drinking, and a high-fat diet worsen DKD through PCD. Research indicates that the combination of nicotine and a high-fat diet can cause oxidative stress and apoptosis of cardiomyocytes by inhibiting AMPK activity and activating caspase-2 signaling pathway, and blocking nicotine receptor can completely prevent this effect (164). Fricker ZP et al. included 2482 participants from the Framingham Heart Study, and found that a high-fat diet led to liver steatosis, which was linked to increased levels of systemic inflammatory and oxidative stress markers, including high-sensitivity C-reactive protein and IL-6 (165). In conclusion, we found no direct evidence that poor lifestyles lead to DKD deterioration through PCD. However, unhealthy lifestyle choices can increase inflammation and oxidative stress, potentially leading to the apoptosis of renal cells.

### 6.2 Traditional Chinese medicine in the prevention and treatment of DKD

#### 6.2.1 Traditional Chinese herbal monomer

##### 6.2.1.1 Targeting apoptosis

Traditional Chinese medicine (TCM) has the potential to safeguard renal function and slow down the progression of DKD by regulating PCD signaling pathways. Catalpol (the main active component of Dihuang [Rehmannia glutinosa]) and morroniside (the main active component of Maqiangan [Cornus officinalis]) synergistically inhibit podocyte apoptosis in DKD by targeting the advanced glycation end product receptor signaling pathway (166). Astragalus polysaccharide promotes the survival of high-glucose-induced RTECs, maintains mitochondrial function, inhibits mitochondria-mediated apoptosis, and improves the condition and activity of the kidney cells through the AMPK/SIRT1/PGC-1α pathway (167). Plantainoside is a benzyl propane molecule derived from Plantago asiatica, and it alleviates cell apoptosis and mitigates DKD in HBZY-1 cells by inhibiting the Akt/NF-κB signaling pathway (167). (**Figure 1**)

##### 6.2.1.2 Targeting pyroptosis

NLRP3, and targeting the activation and formation of NLRP3 has great potential in the treatment of DKD. There are currently several pharmacological therapies targeting NLRP 3. For example, sorasone base (SS), which alleviate HG-induced pyroptosis and oxidative damage by regulating the Nrf2/NLRP3 signaling pathway. Studies have demonstrated that geniposide, an active compound found in the Chinese medicinal herb Gardenia, effectively inhibits oxidative stress and inflammatory reactions, and reduces levels of Caspase-1, IL-1β, and NLRP3 (**Figure 2**). Additionally, geniposide can attenuate pathological GBM thickening induced by hyperglycemia and suppress inflammatory cell infiltration, thereby impeding the advancement of DKD. The underlying mechanism behind these effects may be associated with the AMPK/SIRT1/NF-κB pathway (168).Total flavonoids of Abelmoschus manihot (TFA) (medicinal corolla with stamens and styles) modify modification-mediated NLRP3 inflammasome activation and PTEN/PI3K/Akt signaling by targeting N6-methyladenosine (m6A). Fucinose gum (FPS) suppresses podocyte pyroptosis mediated by the NLRP3 inflammasome by regulating the AMPK mTORC3/NLRP1 signaling axis in DKD, thereby relieving DKD. Carnosine, a dipeptide composed of β -alanine and L-histidine, has shown great potential for targeting caspase-1 to inhibit podocyte pyroptosis in DKD. Carnosine was found to significantly reverse albuminuria and histopathological changes in STZ-induced diabetic mice, and to alleviate renal inflammation and pyroptosis. Tanshinone IIA is one of the main components of Danshen root, which slows the progression of DKD by regulating the Txnip/NLRP 3 inflammatory body, inhibiting the expression of ROS and superoxide anion, thus inhibiting the pyroptosis caused by oxidative stress (169). Moreover, Tan IIA also inhibited pyroptosis in HRGEC cells by inhibiting the activation of the NLRP 3 inflammasome.; Salidroside suppresses activation of NLRP 3 inflammasome involving Caspase-1, thereby alleviating high glucose-induced ECM deposition in mouse mesangial cells; paclitaxel, a bioactive compound derived from Taxus, alleviates DKD in palmitate-induced damage in podocytes by reducing the expression of TGF- β1 (170).

TCM compound ingredients are complex and characterized by multi-component components, Zuogui-Jiangtang-Yishen decoction (ZGJTYS) is a traditional Chinese medicine compound for clinical treatment of diabetic nephropathy. It inhibits intestinal-derived TMAO through the mROS-NLRP 3 axis, and slows DKD by reducing podocyte damage. Tangshen Formula suppresses the activation of NLRP 3 inflammasome by acting on the ROS-Txnip axis, thus alleviating kidney injury caused by DKD. (**Figure 2**)

##### 6.2.1.3 Targeting ferroptosis

CalycosinCalycosin (C16H12O5), an isoflavone compound derived from the dried root of Huangqi (Astragalus membranaceus), a traditional Chinese medicinal herb, exhibits immunomodulatory, anti-inflammatory, antiviral, and antioxidant properties. Researchers have discovered that calycosin reduces lipid ROS and free ionic iron intake in HK-2 cells, thereby preventing cell death induced by iron under high glucose conditions (171). Quercetin improves diabetic renal injury by inhibiting iron death by activating the Nrf 2/HO-1 signaling pathway (172). Vitexin, an active ingredient derived from mountain mustard, mung bean, passion flower, and other natural plants, can increase GPX4 expression by activating the Nrf2/HO-1 pathway and further inhibit lipid peroxidation and iron death. Mangiferin monosodium salt (MGM) upregulates mevalonate (MVA)-mediated antioxidant capacity (GPX 4 and FSP 1/CoQ 10/NADPH) and impairs ACSL 4-mediated lipid driver production in the kidney (173). ACSL4 The strongest inhibitor, rosiglitazone (Rosi), improved renal function in DKD mice and reduced the lipid peroxidation products and iron content, thus inhibiting iron death. Platycodon grandiflorum D (PD) treatment inhibited the cell iron death induced by HG, downregulated the expression of ACSL 4 and TFR 1, and upregulated the expression of FTH-1, SLC7A11, and GPX 4 (82, 174). (**Figure 3**)

##### 6.2.1.4 Targeting autophagy

Astragaloside IV, a purified saponin obtained from the root of Huangqi (Astragalus membranaceus), hinders activation of mesangial cells and promotes autophagy by upregulating SIRT1 expression while downregulating NF-κB p65 acetylation. Consequently, it effectively impedes the progression of DKD (175). Icariin is a TCM extract known for nourishing the kidney and reinforcing Yang. It can potentially become a novel treatment option for tubulointerstitial fibrosis in DKD by restoring autophagy through the miR-192-5p/GLP-1R pathway (176) (**Figure 4**).

#### 6.2.2 Traditional Chinese medicine formulation

Compared to chemical reagents targeting single molecular targets, TCM formulations exhibit better efficacy in the treatment of DKD. The TCM formulation Qidi Tangshen Granules, developed by Professor Liu Hongfang of our team, upregulates the expression of autophagy-inducing proteins (Atg14 and Beclin1), microtubule-associated protein 1 LC3-II, and downregulates the expression of autophagy substrate transport protein P62, thereby activating autophagy to protect against renal damage (177) (**Figure 4**). Another TCM formulation, Qingre Xiaozheng Yiqi Fang (QRXZYQF), developed by Professor Wang Yaoxian of our team, activates mitochondrial autophagy in DKD to protect podocytes. PTEN-induced kinase 1 (PINK1) and Parkin regulate mitochondrial autophagy, and LC3 and P62 are also involved in this process (178–180)(**Figure 4**). Activated PINK1 recruits and activates Parkin to ubiquitinate mitochondrial proteins, ultimately promoting mitochondrial autophagy (180). We found that the expression of PINK1 and Parkin was decreased in DKD rat models, and treatment with QRXZYQF can reverse this change. These findings suggest that the PINK1/Parkin pathway is triggered in response to QRXZYQF treatment to facilitate the removal of damaged mitochondria. We also observed a decrease in the LC3-II/LC3-I ratio and an increase in P62 levels in DKD models *in vivo* and *in vitro*, indicating impaired mitochondrial autophagy in DKD. Treatment with QRXZYQF can reverse this change, thereby activating autophagy and exerting renal protective effects in DKD (181).

In conclusion, there is some evidence that conventional treatment and TCM therapy have the potential to target PCD in the treatment of DKD (**Table 1**), but more in-depth research is necessary.

**Table 1:** Treatment strategies of modern medicine and traditional Chinese medicine.

Treatment method	Therapeutic	Type(s) of programmed cell death	Therapeutic implications	Reference (s)
Modern medicine	Metformin	Apoptosis, Autophagy	1.Enhancing the formation and subsequent removal of autophagic vesicles through signaling pathways mediated by AMPK and inhibition of mTOR. 2.Inducing renal autophagy through the AMPK/SIRT1-FoxO1 pathway to alleviate kidney damage, improve oxidative stress, and correct glucose metabolism abnormalities. 3.Providing protective effects for the kidneys in DKD by mitigating podocyte loss, apoptosis in mesangial cells, and senescence in tubular cells.	(100, 101, 124)
	SGLT2 inhibitors	Apoptosis, Autophagy, Ferroptosis	1.Activating SIRT1/AMPK, inhibiting Akt/mTOR signaling, and regulating autophagy. 2.Suppresses cell apoptosis and improves fibrosis by upregulating Bcl-2 and downregulating BAX, thereby inhibiting cell apoptosis. 3.Inhibiting cell apoptosis by reducing the expression of pro-apoptotic gene (caspase-3) and increasing the expression of anti-apoptotic gene (Bcl-2) in the kidney and enhancing autophagy by increasing the expression of LC-3 and Beclin-1, thus reducing kidney fibrosis. 4.Preventing the downregulation of the podocyte autophagic mechanism, increase podocyte autophagy levels, and improve podocyte cellular remodeling, thereby protecting podocytes. 5.Improving renal fibrosis in mice by modulating iron homeostasis and regulating the expression of renal oxidative stress-related genes. 6.Reducing serum ferritin levels and improves iron metabolism disorders in patients with type 2 diabetes.	(102–104, 125–127)
	GLP-1RAs	Apoptosis, Autophagy	1.Preventing pancreatic beta-cell apoptosis, suppressing glucagon secretion, possessing antioxidative stress properties, and enhancing endothelial function. 2.Exerting renal protective effects by positively regulating autophagic flux through the AMPK-mTOR signaling pathway.	(105, 109)
	DPP-4 inhibitors	Apoptosis,Pyroptosis	1.Inhibiting inflammation and apoptosis in diabetic nephropathy rats. 2.Exerting kidney-protective effects by antagonizing cell pyroptosis through the attenuation of NLRP3 inflammasome activation (135)	(110, 128)
	nsMRAs	Apoptosis	Reduce the upregulation of proinflammatory mediators, including TNF-α, IL-6, IL-1β, plasminogen activator inhibitor 1 (CXCL1), CCL2, and MCP-1	(157)
	RAS inhibitors	Apoptosis	Ang2 induces oxidative stress via NADPH oxidase and promotes inflammation and fibrosis by activating cytokines such as NF-κB, monocyte chemoattractant protein-1 (MCP-1), and TGF-β. ACEi/ARBs counteract these pathogenic effects.	(149)
Traditional Chinese medicine	Geniposide	Apoptosis	Inhibiting oxidative stress and inflammatory reactions associated with apoptosis, significantly reduceing levels of Caspase-1, IL-1β, and NLRP3 and improving GBM thickening and inflammatory cell infiltration, thereby suppressing the development of diabetic nephropathy.	(111)
	Catalpol and morroniside		Inhibiting podocyte apoptosis in DKD by targeting the advanced glycation end product receptor signaling pathway.	(112)
	Astragalus polysaccharide		Promoteing the survival of high-glucose-induced renal tubular epithelial cells, maintaining mitochondrial function, inhibiting mitochondria-mediated apoptosis, and improveing the condition and activity of the kidney cells through the AMPK/SIRT1/PGC-1α pathway.	(113)
	Plantainoside		Lleviating cell apoptosis and mitigates DKD in HBZY-1 cells by inhibiting the Akt/NF-kB signaling pathway.	(130)
	Fucoidan	Pyroptosis	regulating the AMPK/mTORC1/NLRP3 signaling pathway to inhibit NLRP3 inflammasome activation	(131)
	Tanshinone IIA		inhibiting pyroptosis through the regulation of the Txnip/NLRP3 inflammasome	(140)
	Calycosin	Ferroptosis	Reducing lipid ROS and free ionic iron input in HK-2 cells, inhibiting iron-induced cell death in high glucose conditions.	(115)
	Vitexin		Alleviating diabetic nephropathy by attenuated ferroptosis via activating GPX4.	(132)
	Quercetin		Inhibiting ferroptosis via activating Nrf2/HO-1 signaling pathway.	(133)
	Astragaloside IV	Autophagy	Inhibiting mesangial cell activation and enhancing autophagy by increasing SIRT1 expression and reducing NF-κB p65 acetylation, thereby preventing the progression of DKD.	(116)
	Icariin		restoring autophagy through the miR-192-5p/GLP-1R pathway.	(114)
	Qidi Tangshen Granules (QDTS)		Qidi Tangshen Granules upregulates the expression of autophagy-inducing proteins (Atg14 and Beclin-1), microtubule-associated protein 1 light chain 3-II, and downregulates the expression of autophagy substrate transport protein p62, thereby activating autophagy to protect against renal damage.	(117)
	Qingre Xiazheng Yiqi Fang (QRXZYQF)		Activating mitochondrial autophagy in DKD to protect podocytes through the PINK1/Parkin/LC3/p62 pathway.	(118–120)

## 7 Conclusions

With the increasing understanding PCD, the abundant evidence mentioned above supports the close association of apoptosis, ferroptosis, pyroptosis, and autophagy with the pathogenesis of DKD. Conventional DKD management strategies alone, such as SGLT2 inhibitors, GLP-1RAs, DPP4 inhibitors, are no longer sufficient to meet the current clinical needs. In this review, we summarized multiple PCD-related mediators and regulatory pathways, which greatly contribute to the identification of potential new targets and drugs for DKD treatment. We also summarized the relationship between various clinical treatments of DKD and PCD. These clinical treatment options include both drug therapy and non-drug therapy recommended by the guidelines, as well as TCM therapy that is currently showing efficacy in the prevention and treatment of DKD. However, it must be pointed out that the complex diversity of TCM components has brought continuous challenges, because its specific active pharmacological components and molecular mechanisms of action are still under debate. It is gratifying that TCM and modern medical therapies have the potential to target PCD for the treatment of DKD from the current evidence. Therefore, we call for targeted PCD to carry out drug research and development for the treatment of DKD in the future, and to carry out integrated traditional Chinese and Western medicine research to improve the prognosis of DKD patients.
